# Study of Optimal Replacement of Thyroxine in the Elderly (SORTED) – results from the feasibility randomised controlled trial

**DOI:** 10.1186/s13044-016-0034-x

**Published:** 2016-10-10

**Authors:** Salman Razvi, Lorna Ingoe, Vicky Ryan, Simon H. S. Pearce, Scott Wilkes

**Affiliations:** 1 Institute of Genetic Medicine and Queen Elizabeth Hospital, International Centre for Life, Newcastle University, Central Parkway, Newcastle upon Tyne, NE1 3BZ UK; 2 Department of Endocrinology, Queen Elizabeth Hospital, Gateshead, NE9 6SX UK; 3 Institute of Health & Society, Newcastle University, Baddiley Clark Building, Richardson Road, Newcastle upon Tyne, NE2 4AX UK; 4 Department of Pharmacy, Health and Wellbeing, Faculty of Applied Sciences, University of Sunderland, Sunderland, SR1 3SD UK

**Keywords:** Hypothyroidism, Older age, Feasibility

## Abstract

**Background:**

Hypothyroidism is a common condition, particularly in the older population. Thyroid hormone requirements change with age and serum TSH levels also alter, especially in older patients. However, in practice laboratory reference ranges for thyroid function are not age-specific and treatment in older patients aims to achieve a similar target thyroid function level as younger age groups.

**Methods:**

A dual centre, single blind, randomised controlled trial was conducted to determine the feasibility of a future definitive RCT in hypothyroid individuals aged 80 years or older who were treated with levothyroxine. Potential participants were identified from 17 research-active GP practices (*n* = 377), by opportunistic invitations (*n* = 9) or in response to publicity (*n* = 4). Participants were randomly allocated to either usual (0.4–4.0 mU/L) or a higher (4.1–8.0 mU/L) target serum TSH range. Information on participants’ willingness to enter the trial, acceptability of study design, length of time to complete recruitment and dose titration strategy was collected.

**Results:**

Fifteen percent (57/390) of potentially eligible hypothyroid individuals consented to participate in this trial and 48 were randomised to trial medication for 24 weeks, giving a recruitment rate of 12 %. Recruitment averaged 5.5 participants per month over approximately 9 months. Eight participants withdrew (3/24 and 5/24 in the usual and higher TSH arms, respectively) with the commonest reason cited (5 patients) being tiredness. Interestingly, 3/5 participants withdrew from the site that required a visit to a Research Facility whereas only 5/43 participants withdrew from the site that offered home visits. In the higher TSH arm, of those participants who completed the study, approximately half of participants (10/19) reached target TSH.

**Conclusions:**

It is feasible to perform a randomised controlled trial of thyroid hormones in hypothyroid patients aged 80 or older. A definitive trial would require collaboration with a large number of General Practices and the provision of home visits to achieve recruitment to time and target. Power calculations should take into account that approximately 12 % of those approached will be randomised and 1 in 6 participants are likely to withdraw from the study. Finally, several dose adjustments may be required to achieve target serum TSH levels in this age group.

**Trial registration:**

ISRCTN Number: 16043724 Registered 22 June 2012

Clinicaltrial.gov Number: NCT01647750

EudraCT Number: 2011-004425-27

## Background

Thyroid hormones regulate metabolism and impact on several organs in the body. Hypothyroidism (underactive thyroid) is a common endocrine condition in which the thyroid gland produces insufficient thyroid hormones. Hypothyroidism is more prevalent in women and in older individuals with rates of up to 16 % reported in those aged over 80 years [1, 2]. Hypothyroidism is diagnosed based on results of blood tests with low thyroid hormone levels in the presence of high serum Thyroid Stimulating Hormone (TSH) concentrations (usually > 4.0 mU/L). Treatment with the synthetic form of thyroxine (Levothyroxine or LT4) is the treatment of choice in these patients and patients are generally managed in primary care. LT4 is prescribed to 3–4 % of the general population and its use is increasing [3–5]. Thyroid function changes with age and several reports suggest that the upper limit of the TSH reference range increases with age [6–10]. In addition, observational studies have shown that older individuals with a slightly raised serum TSH level have no adverse consequences [11–16]. In fact, one report suggests that a slightly raised TSH may be beneficial for survival in 85-year old individuals followed up for four years [17]. Despite this, all patients are treated uniformly with the aim of achieving a serum TSH target level (usually 0.4–4.0 mU/L) and age-specific ranges are not used. Moreover, evidence obtained from General Practice records suggests LT4 is increasingly being prescribed for older individuals and for minimally raised serum TSH levels [18]. This issue is of increasing importance as the populations in most developed – and many developing – countries are ageing and diagnoses of hypothyroidism are therefore likely to rise. Before a definitive randomised controlled trial (RCT) in the older hypothyroid population can be conducted it would be highly desirable to assess its feasibility. We, therefore, designed a feasibility RCT to investigate lower dose LT4 (aiming for a higher than usual serum TSH) versus usual dose LT4 (aiming for the currently utilised serum TSH range) in hypothyroid individuals aged 80 years or more. We report the feasibility of this trial in this paper.

## Methods

The detailed protocol has been published previously [19]. Briefly, a dual-centre single-blind RCT of elderly (≥80 years) individuals with primary hypothyroidism and good biochemical control (as demonstrated by serum TSH levels within the reference range in the preceding 3 months) was organised. After providing written informed consent, participants were randomised in a 1:1 ratio to either usual dose LT4 (aiming to continue to keep serum TSH between 0.4 and 4.0 mU/L) or lower dose LT4 (reduced by 25 mcg daily initially and a further 25 mcg daily, if required at 12 weeks) to aim for a slightly higher serum TSH level of 4.1–8.0 mU/L. After randomisation participants were assessed at 12 and 24 weeks, with a final follow-up phone call at 25 weeks. The inclusion criteria required participants of either gender, aged 80 years or older, living independently in the community, who had been prescribed LT4 (at least 50 mcg daily) for at least 6 months and whose serum TSH was between 0.4 and 4.0 mU/L in the preceding 3 months. Patients were excluded if they were deemed to not have capacity to provide written informed consent, had severe chronic medical conditions that would prevent participation, thyroid cancer, on medications affecting thyroid function, non-English speakers, had participated in another RCT in the previous 3 months or had lactose intolerance.

The primary objectives were to demonstrate that recruitment to such a trial is possible; to gauge participants’ acceptability of being part of the study; to assess the length of time required to complete recruitment; to assess the dose titration strategy and the length of time required to achieve desired TSH levels; and to gauge medication compliance.

The design and analysis followed published recommendations for feasibility studies [20, 21]. As such, no formal sample size calculation was performed; a target sample size of 50 randomised participants was deemed sufficient to assess the feasibility of the trial. The data analyses were descriptive, and statistical comparisons between randomised treatment groups were not undertaken.

## Results

### Screening and recruitment

**Fig. 1:**
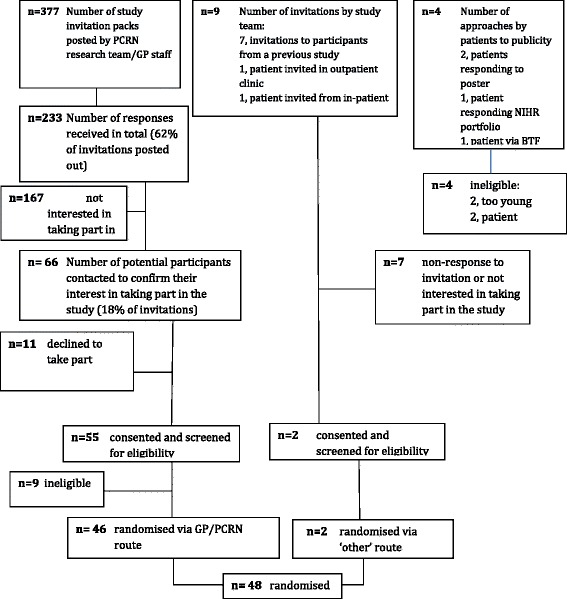
Recruitment summary

Participants were screened for their eligibility to participate in the trial via one of three possible routes: from participating GP practices, hospital endocrine clinics or self-referral responding to posters in hospitals and participating GP practices. (See Fig. 1)

Twenty research-active practices agreed to identify potential patients for the trial. These GP practices were identified by the then National Institute for Health Research, Primary Care Research Network. Of these, two practices did not have the capacity to identify patients for the study and a third responded late in the recruitment process. The remaining 17 GP practices facilitated the identification of potential participants and posted letters of invitation (Table 1). The prevalence of treated hypothyroid patients aged ≥ 80 years ranged from 0.5 to 1.4 % of the total number of patients registered in those practices (Table 1). A total of 377 invitation letters (with a reply slip and stamped addressed envelope) were sent by practice staff and 233/377 (62 %) responded, of which 66 (18 % of total invitations) agreed to participate. The protocol allowed the participating GP practices to send reminder letters to patients that had not replied but this was not followed. Subsequently, 11 individuals changed their mind and declined participation. The reasons given for changing their mind related to either not understanding the study requirements or due to change in circumstances (such as illness). A further 9 patients were deemed ineligible after study-specific screening as either their serum TSH was too low (*n* = 6) or too high (*n* = 3). Therefore, a total of 46 patients (12 % of the 377 potentially eligible participants) were randomised via this route.

**Table 1:** Number of participants approached and randomised by each GP practice

Practices ^a^	Total registered patients (n) (A)	Potentially eligible hypothyroid patients aged ≥ 80 year (% of A)	Invites sent ^c^ (n) (B)	Replies received (% of B)	Consented to be screened (n) (% of B)	Randomised (n) (% of B)
A	16,340	112 (0.7 %)	112	63 (56 %)	20 (18 %)	13 (12 %)
B	5475	42 (0.8 %)	42	25 (60 %)	6 (14 %)	6 (14 %)
C	8830	75 (0.9 %)	14	14 (100 %)	7 (50 %)	5 (36 %)
D	9230	58 (0.6 %)	39	23 (59 %)	5 (13 %)	3 (8 %)
E	11,019	63 (0.6 %)	20	15 (75 %)	3 (15 %)	3 (15 %)
F	10,141	28 ^b^	11	10 (91 %)	4 (36 %)	3 (30 %)
G	5960	82 (1.4 %)	20	14 (70 %)	4 (20 %)	3 (15 %)
H	6875	52 (0.8 %)	10	6 (50 %)	3 (30 %)	3 (30 %)
I	4478	57 (1.3 %)	4	4 (100 %)	2 (50 %)	2 (50 %)
J	7460	36 (0.5 %)	36	20 (50 %)	6 (17 %)	1 (3 %)
K	3728	7 ^b^	7	4 (57 %)	1 (14 %)	1 (14 %)
L	11,788	31 ^b^	20	14 (70 %)	1 (5 %)	1 (5 %)
M	6893	9 ^b^	9	3 (33 %)	1 (11 %)	1 (11 %)
N	19,223	20 ^b^	10	7 (70 %)	1 (10 %)	1 (10 %)
O	3274	3 ^b^	3	1 (33 %)	1 (33 %)	0 (0 %)
P	3296	12 ^b^	5	2 (40 %)	1 (20 %)	0 (0 %)
Q	5565	15 ^b^	15	8 (53 %)	0 (0 %)	0 (0 %)
Total	139,575	703	377	233 (62 %)	66 (18 %)	46 (12 %)

^a^The names of the GP practices are not shown to protect identity

^b^For these practices the total number of potentially eligible patients was not provided

^c^Some practices were asked to only send invites to a random selection of patients to be able to obtain a wide spread of responses from GP practices as possible

Another 13 participants were invited by either the study team or self referred in response to publicity posters in hospitals, the NIHR portfolio or via the patient-led charity the British Thyroid Foundation newsletter. Of these, 2 (15 %) participants were screened and subsequently randomised.

### Randomisation process

Participants were randomised in a ratio of 1:1, using random permuted blocks. Randomisation was stratified by usual (pre-study) LT4 dose (50, 75, 100 and 125 mcg daily) and administered by the Newcastle Clinical Trials Unit using a secure password-protected web-based system (Table 2).

**Table 2:** Distribution of patients by randomisation strata (pre-study dose of Levothyroxine) and allocated treatment group

Pre-study dose of Levothyroxine	Treatment group		Total randomised
	Target TSH range 0.4–4.0 mU/L	Target TSH range 4.1–8.0 mU/L	
50 μg daily	5	5	10
75 μg daily	9	9	18
100 μg daily	8	7	15
125 or more μg daily	2	3	5
Total	24	24	48

### Baseline characteristics

The median age of the participants was 83 years (range: 80 to 93 years) and majority (71 %) were women (Table 3).

**Table 3:** Baseline characteristics

	Total *n* =48
Sex	
Female (n,% of females)	34 (71 %)
Male (n, % of males)	14 (29 %)
Age (years) Median (range)	83.0 (80.0 − 93.0)
Blood pressure (mmHg) Median (IQR)	
Systolic	154.5 (141.0 − 168.5)
Diastolic	85.0 (77.0 − 92.5)
Physical examination Mean (sd)	
Height (cm)	158.8 (8.8)
Weight (kg)	68.5 (13.1)
BMI (kg/m^2^)	27.1 (4.7)
Pulse (bpm)	67.9 (9.8)
Chronic conditions n (%)	
Type 2 diabetes mellitus	4 (8.3)
Hypertension	24 (50)
Ischaemic heart disease	13 (27.1)
Cerebrovascular disease	7 (14.6)
COPD	6 (12.5)
Blood results Median (IQR)	
TSH (mU/L)	1.43 (0.88 − 2.64)
FT4 (pmol/L)	18.8 (16.7 − 19.7)
FT3 (pmol/L)	3.8 (3.6 − 4.2)
Total Cholesterol (mmol/L)	4.9 (4.4 − 6.2)
HDLc (mmol/L)	1.6 (1.5 − 1.9)
Triglycerides (mmol/L)	1.3 (1.0 − 1.9)
Serum CTX (pg/mL)	0.27 (0.17 − 0.37)
TPO antibodies	
< 35 IU/ml (n, %)	28 (58 %)
≥ 35 IU/ml (n, %)	20 (42 %)

*IQR* interquartile range, *sd* standard deviation, *bpm* beats per minute, *COPD* chronic obstructive pulmonary disease, *TSH* thyroid stimulating hormone, *FT4* free thyroxine, *FT3* free triiodothyronine, *HDLc* high density lipoprotein cholesterol, *CTX* carboxy-terminal collagen crosslinks, *TPO* thyroid peroxidase

### Primary outcome measures

This feasibility trial had five primary outcome measures.

1. Participants’ willingness to enter the trial (as gauged by the ratio of those who consented to participate to those who were potentially eligible and approached): 57 out of 390 (15 %) potentially eligible patients consented to participate but, as stated above, 9 individuals (16 % of the potentially eligible group) were not eligible due to abnormal thyroid function at screening.
2. Participants’ acceptability of study design (as measured by the completion rate of participants in each randomised group): 21 out of 24 (87.5 %) and 19/24 (79.2 %) randomised participants completed the trial in the usual and higher TSH arms, respectively.
3. Participant recruitment rate (as measured by the number of patients randomised divided by the length of the recruitment period): Participants were recruited between 17^th^ October 2012 and 10^th^ July 2013 with the first participant randomised on 9^th^ November 2012 and the last one on 10^th^ July 2013. The projected recruitment was 5 or 6 participants/month. Over the 267 days that trial recruitment was open 48 participants were recruited (5.5 participants/month) (See Fig. 2).

**Fig. 2:**
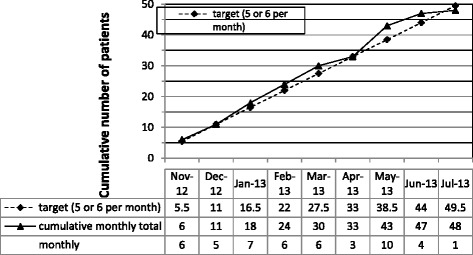
Plot of cumulative number of patients randomised, target versus actual
4. Dose titration strategy and length of time required to achieve desired TSH levels (as calculated by number of participants in each group that reach target TSH range at both 12 and 24 weeks): For the 40 participants who remained in the study until their final study assessment, most participants in the usual dose arm stayed within their target TSH range of 0.4–4.0 mU/L whereas half of the higher target TSH arm achieved their target TSH range (Table 4).

**Table 4:** Dose titration strategy and compliance with study medications

Dose titration strategy: Participants who remained in the study until their final study assessment (i.e. excluding withdrawals), *n* = 40	Usual TSH range (0.4–4.0 mU/L) *n* = 21	Higher TSH range (4. 1–8.0 mU/L) *n* = 19
Percent achieving target TSH range at week 12 (visit 2)	18/21 (85.7 %)	6/19 (31.6 %)
Percent achieving target TSH range at week 24 (visit 3)	18/21 (85.7 %)	10/19 (52.6 %)
Percent compliant at week 12 (visit 2)	21/21 (100 %)	18/19 (94.7 %)
Percent compliant at week 24 (visit 3)	21/21 (100 %)	18/19 (94.7 %)
5. Medication compliance (tablet count at each visit): At the end of visits 2 and 3, participants were asked to return any surplus study drug in the original packaging to the study team, who verified and documented compliance. Compliance was deemed to be generally good. In participants who completed the trial, *n* = 40, compliance was 100 and 95 % at both visits in the usual TSH and higher target TSH arms, respectively (Table 4).

Adverse Events (AEs) and Serious Adverse Events (SAEs): There were 119 AEs reported by 37 patients; 16 patients in the usual TSH arm reporting 49 AEs (mean = 3.1 per patient, sd = 2.1) and 21 patients in the higher TSH arm reporting 70 AEs (mean = 3.3, sd = 2.2). Patients reported between 1 and 10 AEs over the course of the study. The frequency of AEs with regards to the various organ-systems appeared to be broadly similar. For example, the frequency of participants reporting new-onset or worsening tiredness/fatigue was 41.7 % vs 50 % in the usual and higher TSH arms, respectively. Muscle aches and pains (29.2 % vs 16.7 %), dizziness (20.8 % vs 25 %) and constipation (16.7 % vs 29.2 %) were comparable in the two groups.

There were three SAEs, two in the usual TSH arm and one in the higher TSH arm. (See Table 5 for details).

**Table 5:** Chronological listing of serious adverse events

Treatment allocation	Randomisation date	Date of initial report	Description	Onset Date	Severity	SAE reason	Outcome
Usual TSH arm	01/03/2013	26/04/2013	Stroke	22/04/2013	mild	Involved patient hospitalisation	Recovered with sequelae
Usual TSH arm	20/03/2013	13/06/2013	Possible overdose on levothyroxine	12/06/2013	mild	Other significant medical event	Completely recovered
Higher TSH arm	21/01/2013	01/07/2013	Cardiac arrest due to acute myocardial infarction	25/06/2013	severe	Life threatening	Death (after study completion: 05/08/2013)

Site of follow-up: At baseline 71 % of patients (34/48) chose to have their first study visit at home and 29 % (14/48) chose to come into hospital out-patients. At visit 2, four patients switched from out-patient visits to home visits with one patient switching in the other direction (this patient had become a hospital inpatient). Of the 40 patients still in the study at visit 3, 80 % (32/40) had their visit at home and 20 % (8/40) had their visit as an out-patient.

Phone contacts: Over the course of the 24 week study period a total of 27 telephone contacts were made by 20 participants and discussed with the research staff. The majority (20/27) were made in the first 12 weeks; of which 15 were in the first 4 weeks. The queries were with regards to new or existing symptoms (20 contacts by 14 participants; *n* = 8 in usual TSH arm and *n* = 6 in higher TSH arm), query relating to study drug (4 phone calls by 3 participants, all in usual TSH arm), and one phone call each for taking study drug in addition to usual dose levothyroxine (usual TSH arm) and problems in swallowing study drug (usual TSH arm).

Withdrawals and reasons: Eight patients withdrew from the study; three from the usual TSH arm and five from the higher TSH arm (Table 6). Five patients withdrew before visit 2 (two usual TSH, three higher TSH), one patient withdrew at visit 2 (higher TSH arm), and two patients withdrew after visit 2 but before visit 3 (one usual TSH and 1 higher TSH arm, respectively). Three of the five participants withdrew (60 %) from the site that required a visit to a Research Facility whereas only 5/43 participants (12 %) withdrew from the site that offered home visits. In seven participants, the reasons cited for withdrawal were AEs (mainly tiredness or constipation in six and infected foot and wrist pain in a seventh). One participant was withdrawn from the study by the research team for safety reasons as she was found to be taking the study drug in addition to her usual levothyroxine medication.

**Table 6:** Chronological listing of withdrawals

Subject ID	Treatment allocation	Randomisation date	Withdrawal date	Days in the study	Reason (severity and relation to study drug)
709	Reduced dose	07/12/2012	09/01/2013	34	Withdrew due to AEs experienced whilst on the study: constipation, tiredness and generally unwell (mild, possibly related)
103	Usual dose	11/01/2013	23/01/2013	13	Withdrew due to “feeling unwell” (mild, possibly related)
104	Reduced dose	11/01/2013	27/03/2013	76	Withdrew due to infected right foot (moderate, not related), loose stools (moderate, unknown relationship to study drug) and sore wrist (mild, unknown relationship to study drug)
509	Reduced dose	26/11/2012	22/04/2013	148	6 weeks after Visit 2 withdrew from the study due to tiredness (moderate, possibly related)
205	Usual dose	01/03/2013	22/04/2013	53	Withdrew due to fatigue which started 2 weeks after commencing the study drug. The patient was hospitalised with a mild stroke on 22/04/13, reported to be linked to ongoing hypertension (severe, not related).
515	Reduced dose	18/02/2013	08/05/2013	80	Withdrew after experiencing several AEs: dry skin, dry hair, feeling cold, weight gain and tiredness (mild, possibly related), and swollen face with itching (mild, not related) - unscheduled home visit: thyroid function normal, weight gain of 0.3kgs.
521	Reduced dose	15/05/2013	12/06/2013	29	Withdrew after experiencing several AEs: nausea and loss of appetite (mild, not related), vertigo (mild, not related) and confusion (mild, not related)
614	Usual dose	20/03/2013	27/06/2013	100	At Visit 2 patient reported that she had been taking her prescribed dose of LT4 as well as the study medication. Patient reported no AEs. Serum thyroid function was normal (TSH 0.60, FT4 20.8). Subsequently the patient changed her mind and withdrew.

*AEs* adverse events, *LT4* levothyroxine

## Discussion

It is currently unclear what the best practice for managing hypothyroidism is in older people. There is good evidence that thyroid hormone requirements change with age and that the current practice of treating everyone in a uniform fashion may not be appropriate. This feasibility RCT has demonstrated that reducing the dose of the synthetic thyroid hormone levothyroxine is possible and that patients are willing to participate.

The population of the western world - including the United Kingdom - is ageing. Ageing of the population refers to both the increase in the median age as well as the increase in the absolute number and proportion of older individuals. The number of people aged 75 and over has increased by 89 % over the period 1974 to 2014 and now makes up 8 % of the total population [22]. Between 2015 and 2020, when the general population is expected to increase by 3 %, it is estimated that people aged 65 years or more will rise by 12 % to 1.1 million; those over 85 years by 18 % to 300,000; and the number of centenarians by 40 % (7000) [22]. Similar demographic changes are occurring across much of the Western world. For instance, in the United States it is estimated that there are now about six million persons aged 85 years and older and this number may reach 19 million in 2050 [23]. Data from this feasibility study suggests that the prevalence of treated hypothyroidism in the over 80 age group is between 0.5 and 1.4 % of the entire population. It is also apparent that the majority of hypothyroid patients in this age group are on modest doses of LT4 – which is indicative that treatment was commenced for borderline raised TSH levels. This has significant implications for the management of hundreds of thousands of older hypothyroid individuals in the United Kingdom. Furthermore, as the number of older individuals increases then the absolute number of older people with treated hypothyroidism is also likely to grow.

The result of this feasibility trial has shown that it is possible to recruit participants into an interventional trial to aim for a higher than usual target serum TSH. The randomisation rate was approximately 5.5 participants per month from the 17 GP practices that identified patients and from the other sources of referral. Overall, 12 % of potentially eligible patients consented to participate and were randomised. This feasibility data suggests that a large-scale RCT would have to recruit from hundreds of GP practices. Furthermore, allowance would have to be made for 16 % (possibly even higher in a study of a longer duration) of participants to withdraw from the study. Any subsequent full RCT would have to be of longer duration (probably several years) and designed to be able to answer the important question of the optimum target serum TSH to aim for in older hypothyroid patients. Such a trial would require hard clinical end points including cardiovascular events and fractures as its main outcome measures.

One of the limitations of this trial is that we are unable to assess the characteristics of individuals who refused to participate. This is because study invitations were sent by individual GP practices and the responses were received in an anonymised form by the study team. Therefore, it is not possible to assess differences between responders and non-responders.

Adverse events and serious adverse events did appear to be similar in both the usual TSH arm as well as the higher TSH arm in our study. However, this feasibility trial was not powered to detect a significant difference in any effect size. It is therefore possible that there may be a higher incidence of adverse events in a larger trial over a longer follow-up period. This would require support to be available for study participants. In this trial telephone support was made use of by a number of participants and may have contributed to their retention. The telephone contacts were made mostly in the first few weeks after randomisation and related to either symptoms or study drug. It is important to consider providing telephone support in a full large RCT particularly in the first 4 weeks after commencing study drug. In addition, it is also evident that participants in this age group prefer to have home visits rather than to have to come to a hospital research facility. It is unclear how participants would view visits to their local GP practice.

It is important to consider strategies that would help retain participants in the trial and lead to high completion rates. Approximately 4 out of 5 randomised participants completed this trial. This was achieved by offering flexible study dates, times and venue, providing alternative appointments at short notice, being seen by the same member of staff at each visit and availability of telephonic support.

This feasibility trial revealed that of the patients randomised to a higher TSH range, who remained in the study until their final study assessment, only half reach their target. This is not surprising given that up to half the hypothyroid population, irrespective of age does not have good biochemical control as evidenced by their serum TSH level [24, 25]. The implications of this are that more frequent or smaller dose adjustments of levothyroxine may be required in the full trial to be able to achieve a higher proportion of participants in the desired relaxed TSH range.

## Conclusion

In conclusion, this feasibility trial has shown that it is possible to recruit and retain patients with levothyroxine treated hypothyroidism aged 80 years or older into a RCT. Several important lessons have been learnt that would help to design a trial that should be able to successfully recruit and retain patients into a longer-term study.

## Abbreviations

AEs: Adverse events
BMI: Body mass index
COPD: Chronic obstructive pulmonary disease
CTX: Carboxy-terminal collagen crosslinks
FT3: Free triiodothyronine
FT4: Free thyroxine
GP: General practitioner
IQR: Interquartile range
LT4: Levothyroxine
PCRN: Primary Care Research Network
RCT: Randomised control trial
SAEs: Serious adverse events
sd: Standard deviation
TPO: Thyroid peroxidase
TSH: Thyroid stimulating hormone
